# Chest Pain of Atypical Cause in a Young Man

**DOI:** 10.3390/diagnostics12081881

**Published:** 2022-08-03

**Authors:** Justyna Fijolek, Dariusz Gawryluk, Dorota Piotrowska-Kownacka, Krzysztof Ozieranski, Romuald Wojnicz, Elzbieta Wiatr

**Affiliations:** 1 The Third Department of Pneumonology and Oncology, National Tuberculosis and Lung Diseases Research Institute, 01-138 Warsaw, Poland; dpgawryluk@gmail.com (D.G.); e.wiatr@igichp.edu.pl (E.W.); 2 The First Department of Clinical Radiology, Medical University of Warsaw, 02-091 Warsaw, Poland; dodo@mrlab.pl; 3 The First Department of Cardiology, Medical University of Warsaw, 02-091 Warsaw, Poland; krzysztof.ozieranski@gmail.com; 4 Silesian Nanomicroscopy Center, Silesia LabMed: Research and Implementation Center, Medical University of Silesia, 41-808 Zabrze, Poland; rwojnicz@sum.edu.pl

**Keywords:** granulomatosis with polyangiitis, cardiac magnetic resonance, myocarditis, cardiac biopsy

## Abstract

Granulomatosis with polyangiitis (GPA) is a rare systemic vasculitis that classically affects the upper respiratory tract, lungs, and kidneys. The involvement of other organs occurs but is less frequent. Clinically overt cardiac involvement is rare. We present a rare case of thoracic pain caused by cardiac involvement in GPA, without any other symptoms. The diagnosis was made using an integral approach, with several complementary imaging modalities, including cardiac histology.

**Figure 1:**
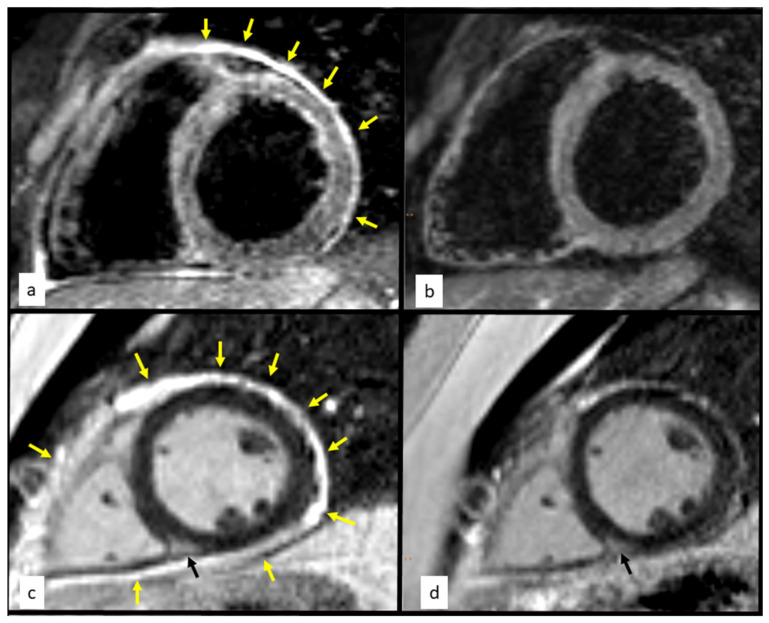
A 25-year-old man was admitted to the National Tuberculosis and Lung Diseases Research Institute in January 2020 for recurring chest pain. In November 2016, granulomatosis with polyangiitis (GPA) involving the ear, nose, throat, lungs, kidneys, and skin was recognized, with the presence of antiproteinase 3 (PR3) antibodies. He was treated with standard immunosuppressive agents (oral cyclophosphamide, 2 mg/kg; and prednisone, 0.8 mg/kg) [1] and achieved complete remission in November 2018, but experienced recurrent chest pain in March 2019. The pain lasted for 4–6 days and recurred every 3–4 months, without shortness of breath, fever, or other symptoms. On admission, the patient was in good condition. His physical examination results were unremarkable, without respiratory or cardiac pathological findings upon auscultation. A complete blood count showed leukocytosis (14,000 cells/μL) with neutrophilia (10,000 cells/μL). Serum biochemistry revealed an elevated C-reactive protein level of 234 mg/ml (CRP; normal range, <5.0 mg/mL) and increased PR3 antibody levels compared with the most recent results from November 2018 (28 >75 U/mL). There was no renal dysfunction or changes in urine sediment. Basic cardiac enzyme concentrations were normal. Computed tomography showed no lung infiltrates or adenopathies. Electrocardiography revealed sinus tachycardia. Transthoracic echocardiography revealed mild hypokinesis of the left ventricular inferior wall and the interventricular septum, with small pericardial effusion (8 mm on the apex side), without evident thickening of the pericardium. Cardiac lesions were characterized using cardiac magnetic resonance (CMR) imaging [2], which showed features of acute pericarditis and myocarditis with a decreased ejection fraction of 45% ((**a**) black blood T2-weighted STIR images in the short-axis plane—global myocardial oedema and thickened pericardium with hyperintense signal (arrows); (**c**) late gadolinium enhancement images in the short-axis plane—small intramural enhancement (black arrows) and thickened, enhanced pericardium (yellow arrows)). Figure 2 and Figure 3. Considering the previous history of GPA and increased antineutrophil cytoplasmic antibody (ANCA) titer, cardiac involvement in GPA was considered. However, the patient showed no other organ symptoms suggestive of vasculitis. Subsequently, he was referred to a cardiology center. The results of coronary angiography testing were normal, and the patient underwent endomyocardial biopsy (EMB). Immunohistochemical examination (2) showed active myocarditis with myocyte damage and infiltration of macrophages (red color); some of them were in tight contact with injured myocytes (arrows) (original magnification: ×400). Masson trichrome staining (3) highlighted the interstitial collagenous connective tissue (blue color) with the concomitant thrombus formation (arrows) (original magnification: ×100). The pathomorphological picture corresponded to infection-negative active myocarditis, suggestive of GPA [3]. Finally, isolated cardiac involvement in GPA was observed (Birmingham Vasculitis Activity Score; BVAS score = 4). Due to vasculitis relapse, rituximab was administered for induction (four weekly infusions of 375 mg/m^2^) [1], followed by oral methotrexate (25 mg per week) as maintenance therapy, which improved the patient’s symptoms and cardiac lesions. Over the 2 years of follow-up, the symptoms did not return, and improvement, according to CMR images, was maintained ((**b**): complete regression of pericardial thickening and myocardial oedema compared to baseline; (**d**): partial regression of pericardial thickening and enhancement compared to baseline).

**Figure 2:**
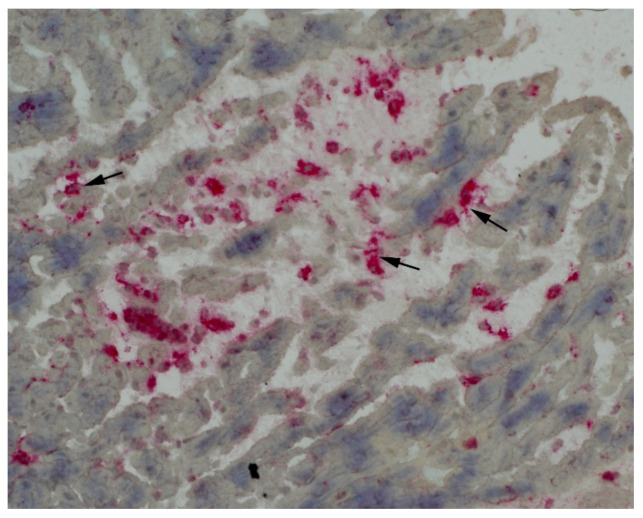
To the best of our knowledge, GPA with the only recurrent symptom being chest pain caused by cardiac involvement has not been reported. In general, clinical signs of cardiac involvement in GPA are rare and commonly associated with coronary artery or pericardial involvement [4]. Echocardiography is usually a safe and easily accessible method, but its sensitivity and specificity are limited [5]. CMR is most accurate in diagnosing cardiac lesions in vasculitis [6] and is reliable for monitoring treatment efficacy [7]. Nonetheless, specific abnormalities in CMR associated with GPA remain undefined. Therefore, despite its invasive nature and limited sensitivity, EMB remains the gold standard for diagnosing myocarditis [8]. In our patient, this procedure was essential for the final diagnosis. Histological documentation of cardiac involvement in GPA is uncommon. In a series of GPA cases, unequivocal cardiac involvement was found in less than 2% of cases [3]. The low diagnostic accuracy of EMB is likely due to the rate of sampling errors [8]. Additionally, the patchy cardiac lesions complicate diagnosis [9]; therefore, both experience and close cooperation between clinicians and pathologists are necessary for accurate diagnosis [9]. In patients with ANCA-positive, multiorgan GPA and histologic findings of granulomatous myocarditis, giant cells, and necrosis, the diagnosis is straightforward [3]. In our patient, the heart was the only organ exhibiting manifestation. Although lacking classic histologic features of GPA, signs of active myocarditis with coronary microvascular diseases in EMB with CMR findings and an increased ANCA titer strongly suggested that GPA was responsible for the histologically verified myocarditis. This was confirmed by favorable outcomes of standard rituximab treatment. Our case emphasizes two important characteristics of GPA. First, limited GPA is a diagnostic challenge. Although EMB may facilitate the diagnosis, the disease cannot be excluded, even lacking typical histologic features. Second, histological examination alone is insufficient for GPA diagnosis and should be interpreted in the clinical context.

**Figure 3:**
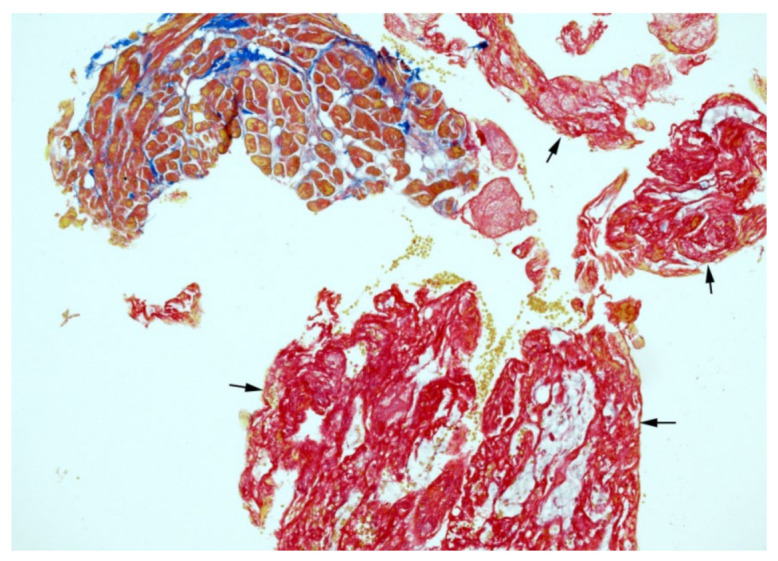
Clinically overt cardiac involvement in GPA is rare (3.3%) [4]; therefore, it may be easily overlooked. Pericarditis is the most common symptom in symptomatic patients (35%) [4], but only a few reports have described myocarditis caused by GPA [10]. Although pericarditis is usually mild, it can relapse or progress to constrictive pericarditis and tamponade [11], whereas myocarditis can lead to congestive heart failure or life-threatening arrhythmia [12]. Cardiac involvement in GPA increases mortality and the risk of relapse [13,14]. Therefore, the possibility of this condition should be considered, particularly when chest symptoms are present. However, cardiac involvement may be clinically silent [15]; therefore, CMR monitoring should be recommended in all GPA patients, irrespective of symptoms or abnormalities on basic cardiac tests. In conclusion, we present a rare case of thoracic pain caused by cardiac involvement in recurrent GPA, without any other symptoms. The diagnosis was made by combining several complementary imaging modalities, including cardiac histology. This case demonstrates the great challenge in diagnosing GPA, particularly in cases with single-organ involvement. It shows that EMB may facilitate the diagnosis, although histological examination is not sufficient enough to establish a GPA diagnosis on its own, especially when lacking typical histologic features. In presenting this case, we would like to emphasize the need for cardiac monitoring in the entire GPA population. Recognizing cardiovascular complications is important because they are linked to increased mortality and impact treatment decisions. Close cooperation between radiologists, pathologists, and clinicians is necessary to establish an appropriate diagnosis.
